# DualFusionNet: A fusion-based dual architecture for visual quality control on fabric surfaces

**DOI:** 10.1371/journal.pone.0346708

**Published:** 2026-04-29

**Authors:** Christopher Mai, Luca Eisentraut, Felix Waigner, Pascal Penava, Ricardo Buettner

**Affiliations:** Chair of Hybrid Intelligence, Helmut-Schmidt-University/University of the Federal Armed Forces Hamburg, Hamburg, Germany; Instituto Politecnico Nacional, MEXICO

## Abstract

Zippers are essential in fashion and beyond. Found on jackets, shoes, and bags, they provide a versatile and practical opening and closing mechanism that seamlessly integrates into everyday use and specialized applications. The quality control of zippers is often still based on a subjective visual inspection by experienced persons. This step is time-consuming, inefficient, and associated with a higher error rate. To address these challenges, the deployment of image processing systems for the automated inspection of zippers is becoming increasingly crucial. To determine whether defects are present, approaches often use architectures that perform local feature extraction. However, there is a risk that global relationships will be overlooked, which must not be ignored in the case of defects. The aim of this study is therefore to improve the detection accuracy of zipper defects by integrating both local and global characteristics into one architecture. To achieve this aim, DualFusionNet is proposed, a dual architecture that combines the advantages of CNN- and Transformer-based feature extraction. The integration of the Adaptive Feature Pyramid Network (AFPN) with a Squeeze-and-Excitation (SE) block facilitates the fusion of features from both architectures, enabling the dual architecture to incorporate local and global relationships for improved classification performance. The efficacy of this approach is evidenced by the attainment of an accuracy of 99.74% and a balanced accuracy of 99.58%. The high accuracy shows that the use of deep learning systems for the automatic inspection of defects in zippers is an effective economic step for companies, as it leads to cost and time savings in production due to the high classification performance. This study provides an overview of the results achieved and other key performance indicators of the architecture used.

## Introduction

Zippers are an omnipresent feature of our lives that most people are familiar with [1]. They are also found in many everyday items that people use regularly, especially in the clothing industry, where zippers are commonly used on jackets, jeans, bags, and suitcases [1]. In addition to their use in the clothing industry, zippers serve practical purposes in numerous other products, providing a reliable solution for opening and closing mechanisms [2]. Their versatility makes them suitable for everyday use, which underlines their importance. However, zippers also highly relevant from a manufacturing perspective. They are a high-volume industrial product that is produced at scale, where extremely low production costs, automated mass production, and susceptibility to errors are essential [2]. Related defects may occur in the fabric or on the zipper tape during processing steps. The former can range from tape indentation defects to large broken taps to small defects within the tape; related defects of the latter include broken teeth or squeezed teeth, for example. Therefore, due to the combination of their relevance to production and the variety of possible defects, it is essential to conduct high-performance quality checks. From an economic perspective, attaching zippers is a relevant step in the process [3], which is why defective ones can lead to rework or scrap and thus increased costs. Undetected defects can have a significant impact on perceived quality.

**Fig 1:**
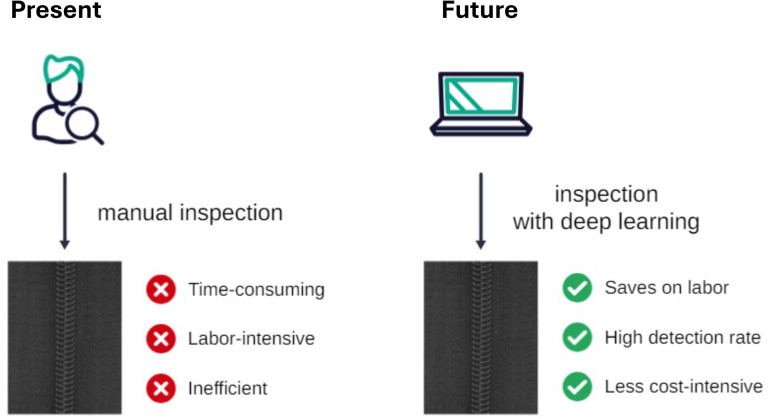
Comparison of the present manual quality inspection process in zipper [13] production and its potential future evolution, which could offer significant advantages through the integration of modern deep learning methods.

Due to the strict quality standards of zipper products and the technical challenges of quality inspection, current zipper inspections rely heavily on subjective visual checks by experienced inspectors, which is highly subjective [4], and suffer from high labor intensity, inefficiency, high rates of missed defects and false positives due to human error and fatigue, while also failing to provide accurate quantitative statistical results necessary for analyzing defect sources [5]. A number of traditional surface defect inspection algorithms have been developed with the objective of addressing the challenge of defect detection. These algorithms can be classified into four main categories [6]: statistical, spectral, model-based, and learning-based. Despite partial success, these methods are limited by their heavy reliance on human-designed features, which makes them sensitive to changes in application conditions [7]. In recent years, deep learning methods have achieved remarkable success in image recognition and classification. Among the various deep learning models, the convolutional neural network (CNN) is the most widely used one, which is most suitable for the binary classification of defective or non-defective zippers for quality inspection. Fig 1 illustrates the advantages of deep learning-based approaches compared to traditional inspection methods in zipper production. Prior research has demonstrated the efficacy of deep learning techniques in object recognition, particularly in the context of defect detection in zipper [4,5,8–12].

Some studies focus solely on classifying the types of defects in zippers but do not address whether a zipper is defective or not. In addition, a number of studies use non-public datasets, so that comparability is not possible. Often, only accuracy is reported, while other metrics important for quality control, such as balanced accuracy, are considered less frequently. Furthermore, the question of how robust the methods presented are is open, as only few studies use cross-validation for evaluation. Considering the criteria mentioned, the current highest accuracy of 95.93% and balanced accuracy of 93.97% indicate that there is still potential for further optimization. Defects on zippers can occur in varying sizes and shapes, some are fine-grained and barely visible, while others are elongated or spread across larger regions. A single architecture often struggles to capture both the local subtleties and the broader structural patterns that characterize these defects. To address these limitations, we develop an effective dual architecture, DualFusionNet, that achieves a high accuracy and balanced accuracy alongside strong performance in industry-relevant metrics. The proposed architecture combines the strengths of ResNet50, which captures local features, and Swin Transformer V2, which captures global relationships. By using AFPN, the features of both architectures are weighted and fused with each other, and the integrated attention module also highlights the most important regions. To evaluate the novel architecture, we evaluate the approach on the MVTech dataset [13]. This study’s results demonstrate that DualFusionNet, achieving a balanced accuracy of 99.58%, effectively differentiates between defective and non-defective zippers, thereby establishing a new benchmark. Implementing this architecture has the potential to significantly improve the efficiency of automated quality control in the zipper manufacturing industry. This can lead to significant cost savings and the mitigation of potential reputational damage. The main results of this study are as follows:

1. We developed DualFusionNet, a hybrid architecture based on a combination of convolutional neural networks (here: ResNet50) and transformers (here: Swin Transformer V2).
2. The intensive evaluation of the zipper dataset from MVTec [13] shows that DualFusionNet achieves a new benchmark in distinguishing between defect-free and defective zipper images.
3. We show that integrating AFPN and an SE block improves accuracy to 99.74%, balanced accuracy to 99.58% and other key performance indicators such as true positive rate to 99.17%, negative predictive value to 99.64% and F1-score to 99.57%.

Our paper is structured as follows: Section Related Work provides an overview of methods for defect detection on surfaces, related work in relation to defect detection on zippers using deep learning methods. Section Methodology contains information about the deep learning approach used, a description of the deep learning architecture used, followed by a detailed explanation of the training and test process and the dataset used for the evaluation. In section Results, we present the results, followed by the discussion in section Discussion. Finally, section Conclusion presents a summary of the key findings and highlights the study’s contributions.

### Related work

Deep learning-based visual quality control has already been successfully integrated into various industries and different technical forms, whether for unsupervised anomaly detection [14,15], classification [16], or defect segmentation. Building on these general advances, quality control of zippers is also a suitable field for the application of deep learning. Structurally, the problem is similar to, for example, analysis methods in additive manufacturing, where local and global information are also used together by hybrid models for quality assessment [17,18].

A zipper is often comprised of two primary components: a zipper tape and a fabric to which it is attached. Both the zipper tape and the fabric exhibit distinct textures and structures, which makes related work comparable to the field of fabric defect detection. Existing detection methods can be divided into traditional algorithms and learning-based methods, whereby the learning-based methods can be further divided into classical machine learning algorithms and deep learning algorithms [19]. Deep learning methods for defect detection approaches can be divided into three categories: supervised, semi-supervised, and unsupervised learning. It can generally be stated that semi-supervised methods perform worse than supervised learning [20]. However, supervised learning methods tend to perform exceptionally well when the dataset includes a sufficient number of examples for each class, thereby enabling the model to learn more effectively from the comprehensive labeled data [20]. Furthermore, these methods often demonstrate better detection rates in comparison to unsupervised methods [21–23], which is the reason why we focus on this case. This section provides an overview of the basics of transfer learning, traditional defect detection methods, and relevant studies on zipper defect detection.

### Traditional defect detection methods

Traditional algorithms can be divided into four main categories: statistical, structure-based, spectral-based, and model-based algorithms [6]. Statistical approaches [24,25] leverage the spatial distribution of gray values in images. Techniques used in these approaches include gray-level co-occurrence matrices (GLCM), autocorrelation analysis, which evaluates the similarity of pixel values at varying distances, and fractal dimension features, which measure the complexity of image textures [19]. Statistical methods differentiate defective from non-defective regions by analyzing distinct statistical features such as similarity, mean value, variance, and regularity using techniques like auto-correlation and co-occurrence matrix methods, which identify defective regions by detecting outliers in the statistical feature distribution [26]. Statistical approaches struggle to distinguish fuzzy and small defects, as these defects do not significantly alter the average gray level of the fabric image, and they are also less effective in detecting fabrics with complex defect distributions [27].

In structural methods [28–30], defect features are characterized by texture elements. The goal of these approaches is to extract the texture elements of defects to represent the spatial placement rules. Shi et al. [31] propose a method in which a low-rank decomposition of gradient information is combined with a structured graph. The image of tissue defects is divided into defect-free and defective regions. When merging the different regions, an adaptive threshold is used so that defect-free and defective grids do not merge. Finally, the matrix decomposition is calculated from the segmentation results, taking into account the previous defect information. In this way, defect-free regions are attenuated and defective regions are emphasized. However, the disadvantage of these methods is that most of them are sensitive to the shape and size of the defects, and the defect patterns should be aperiodic [32].

Spectral methods involve translating images from the spatial domain to the frequency domain for defect detection, utilizing mathematical operators designed to extract relevant features from frequency components and identify defective areas [26]. Representative methods are the discrete cosine transform [33], the Gabor transform [34], or the wavelet transform [35]. Spectral approaches excel in detecting subtle defects like color changes and remain resilient to noise, but are limited to fabrics exhibiting high textural periodicity and are unsuitable for those with random textures [27].

Model-based methods concentrate on modeling the texture of fabric images, utilizing these models to identify any defects present in the images [26]. Approaches in this category include the autoregressive model, the Gaussian Markov Random Field model, or the Gaussian mixture model [27,36]. The disadvantage of these approaches is that they are computationally intensive and therefore not suitable for real-time detection, and they are less good at detecting defects in smaller areas [27].

### Deep learning methods

In order to benefit from the advantages of transfer learning, we focus on the use of pre-trained architectures for DualFusionNet. According to our research, only a few authors deal with the classification of defective zippers using pre-trained architectures.

In [10], the authors present a method for the CNN to restrict its focus to the relevant area of the defective image during training. They introduce an overlap coefficient into the conventional cross-entropy loss function, whereby the greater the distance from the defect, the greater the loss. Additionally, masks are employed during training. The results are evaluated using an EfficientNet-B0 and a custom-built CNN. This approach yields an accuracy of 98.99% for EfficientNet. In the study of Fang et al. [5], the authors put forth a framework for the detection of defects in zipper tapes. The authors highlight the limitations of current deep learning methods in detecting small-scale defects. Their proposed method addresses this issue by employing a two-step process. Initially, regions of large defects and local context regions containing small-scale defects are identified. Subsequently, small-scale defects are detected from the local context regions. This approach achieves a mean average precision (mAP) of 99.55%.

To recognize the various defects that occur in zippers, Sun et al. [4] used a YOLOV5 network. They were able to show the effectiveness in detecting the individual defects and achieved an overall accuracy between 99–100% for each of the six classes of defects. In the work of Xu et al. [37], a method for detecting defects using image-level labels through a Multiple Instance Learning (MIL) framework is presented. The approach employs a custom CNN and ResNet50 to extract image features, which are then segmented into regions (bags). Each instance within these bags is assigned a defect probability by a fully connected layer. If the highest probability among all instances surpasses a threshold, the image is classified as defective. Buettner et al. [16] conducted a study on the impact of a Gaussian filter. Their investigation utilized five pre-trained architectures Xception, ResNet50, InceptionV3, VGG19, and VGG16. The best performance on the zipper dataset was achieved with VGG19, where images were pre-processed using a Gaussian filter. Among other performance metrics, this architecture attained an accuracy of 95.93% and a balanced accuracy of 93.97%.

Table 1 provides an overview of the pertinent literature. Only a limited number of researchers have directed their attention toward the classification of defective and defect-free zippers. In addition, some studies use non-public datasets, which makes it difficult to compare the results. Moreover, data quality and collection are not ensured by external experts. Additionally, the table shows that other important metrics for quality control, like balanced accuracy, are considered less frequently. Furthermore, the robustness of the methods presented and the results reported remains questionable, as only one study used cross-validation for evaluation. Considering the criteria outlined above, the current highest accuracy of 95.93% and balanced accuracy of 93.97% indicate that there is still potential for further optimization. Considering the limitations identified in previous literature, we develop a novel dual architecture called DualFusionNet, which leverages the strengths of ResNet50 and Swin Transformer V2 to capture both local and global features in order to surpass the current benchmark in zipper defect classification.

**Table 1:** Overview of related studies involving zippers. Includes the reference, the task, information on whether peer-reviewed data sets and cross-validation were used, as well as the accuracy and balanced accuracy achieved.

Reference	Peer-reviewed Dataset	Cross-validated	Accuracy (ACC) and Balanced Accuracy (BACC) (in %)
Fang et al. [5] (2021)	✗	✗	ACC and BACC not reported.
Sun et al. [4] (2022)	✓	✗	ACC: 100.00 (for four classes); ACC: 99.00 (for two classes); BACC: not reported
Fraccaroli et al. [10] (2024)	✓^1^ ‖ ✗^2^	✗	^1^ACC: 95.56; ^2^ACC: 98.99; BACC: not reported
Xu et al. [37] (2024)	✗	✗	ACC and BACC not reported.
Buettner et al. [16] (2025)	✓	✓	ACC: 95.93; BACC: 93.97
**This study**	✓	✓	**ACC: 99.74; BACC: 99.58**

## Methodology

### Model architecture

#### DualFusionNet.

DualFusionNet integrates the strengths of ResNet50 and Swin Transformer V2 to effectively extract both local and global features for defect detection in zipper images. ResNet50 functions as a CNN-based feature extractor, primarily capturing local and regional defect patterns. In parallel, Swin Transformer V2 acts as a secondary transformer-based feature extractor, enhancing the model’s ability to incorporate global contextual information. To effectively merge the feature representations from both architectures and leverage their complementary strengths, an AFPN is employed. Since ResNet50 and Swin Transformer V2 generate distinct feature representations, the AFPN facilitates feature fusion by integrating multi-scale information. Specifically, multi-kernel convolutions with kernel sizes of 1×1, 3×3, and 5×5 are utilized to extract features at different spatial scales, ensuring a comprehensive defect detection framework.

Swin Transformer V2 uses the final feature map *X*_Swin_ obtained after completing the extraction process. This feature map has a lower spatial resolution but a higher channel dimensionality ((B, 1536, 6, 6) compared to the feature maps generated by ResNet50 (stage 1: (B, 256, 48, 48); stage 2: (B, 512, 24, 24); stage 3: (B, 1024, 12, 12); stage 4: (B, 2048, 6, 6)). To enable fusion at each stage, *X*_Swin_ is first resized via bilinear interpolation to match the spatial dimensions of the corresponding ResNet feature map. The resulting spatially aligned representation is denoted as ${\tilde{X}}_{\text{Swin}}$:


$${\tilde{X}}_{\text{Swin}}=\text{Interpolation}(X_{\text{Swin}}\to X_{\text{ResNet}}).$$ (1)

The AFPN uses three convolutional layers with different kernel sizes to capture multi-scale information and to project the ResNet (*X*_ResNet50_) and Swin Transformer V2 (${\tilde{X}}_{\text{Swin}}$) feature maps into a compatible channel space, as both representations differ in their characteristics.


$$X_{conv1}=W_{1\times 1}*X_{ResNet50}$$ (2)

$$X_{conv2}=W_{3\times 3}*{\tilde{X}}_{Swin}$$ (3)

$$X_{conv3}=W_{5\times 5}*(X_{conv1}+X_{conv2})$$ (4)

where *W*_1×1_, *W*_3×3_, and *W*_5×5_ denote the learnable convolution kernels with kernel sizes 1 × 1, 3 × 3, and 5 × 5, respectively. The three resulting feature maps are then fused using softmax weighting. First, a 1×1 convolution with three output channels is applied to the concatenated feature maps to generate a three-channel spatial score tensor *Z*.


$$Z=W_{f}*concat(X_{\text{conv1}},X_{\text{conv2}},X_{\text{conv3}}),$$ (5)

$$\alpha =[\alpha_{1},\alpha_{2},\alpha_{3}]=\text{Softmax}(Z)$$ (6)

where *W*_*f*_ denotes the weights of the 1×1 convolution and $\alpha \in {\mathbb{R}}^{3\times H\times W}$ contains the different weighting factors $\alpha_{i}$ for each individual *X*_*conv*_, ensuring that the sum remains $\Sigma \alpha_{i}=1$. The fused feature map is obtained as:


$$X_{\text{fused}}=\alpha_{1}⊙X_{\text{conv1}}+\alpha_{2}⊙X_{\text{conv2}}+\alpha_{3}⊙X_{\text{conv3}}$$ (7)

Since Swin Transformer V2 and ResNet50 generate distinct feature representations, a SE [38] block with a reduction ratio of *r* = 16 is additionally incorporated to enhance channel-wise attention within the fused features. This allows the model to learn to emphasize important channels while suppressing irrelevant ones, thereby improving the efficiency of feature utilization. The final fused feature map, denoted as ${\tilde{X}}_{fused}$, is defined as follows:


$${\tilde{X}}_{fused}=s⊙X_{fused}$$ (8)

where *s* represents the scaling vector for channel weighting.


$$X_{i+1}^{\text{ResNet}}={\tilde{X}}_{\text{fused}},\;X_{i+1}^{\text{Swin}}=X_{i}^{\text{Swin}}+\text{update}({\tilde{X}}_{fused})$$ (9)

Here, $\text{update}({\tilde{X}}_{\text{fused}})$ denotes the feature enhancement update applied to the Swin Transformer feature map, implemented by a 1×1 convolution that restores the original Swin Transformer channel dimensionality followed by bilinear interpolation to match the spatial resolution of $X_{i}^{\text{Swin}}$. The equation in (9) indicates that ResNet50 and Swin Transformer operate as complementary backbone components, with their extracted features being adaptively fused through the AFPN module. Rather than one backbone dominating the process, both architectures make meaningful contributions to the extracted feature representation. The fused features are then propagated into both backbones, with ResNet receiving them directly and Swin Transformer receiving a feature enhancement update. After progressing through all processing stages, the extracted features from both networks undergo GAP. The resulting features are then flattened into a vector, ResNet50 produces a 2048-dimensional feature vector, while Swin Transformer V2 yields a 1536-dimensional feature vector. After the fusion of these two vectors, the result is a 3584-dimensional feature vector. This vector is then passed through two FC layers to perform the final classification. The first FC layer reduces the dimensionality from 3584 to 1024 units and is followed by ReLU activation function, and a dropout layer to prevent overfitting. The second FC layer assigns the 1024 features to the single output unit.

#### ResNet50.

**Fig 2:**
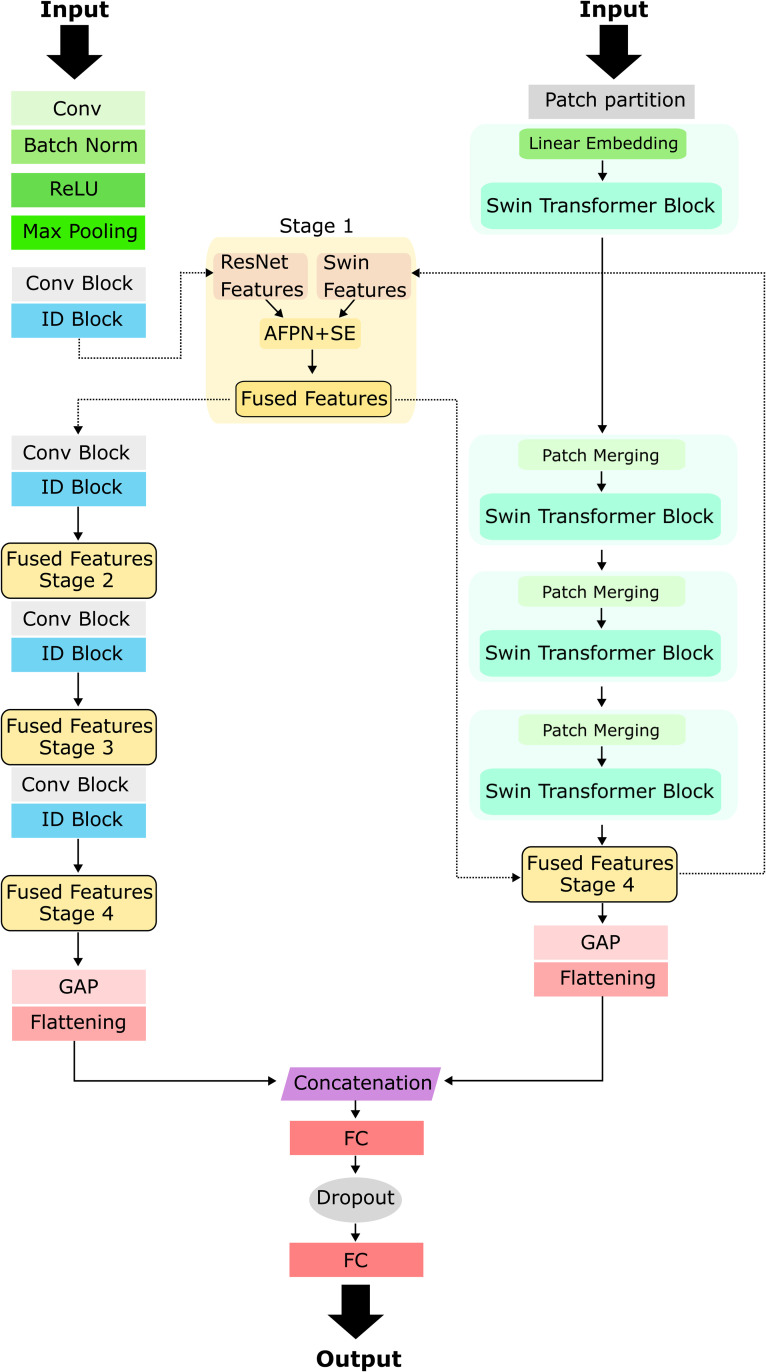
Illustration of DualFusionNet. ResNet50 is depicted on the left and Swin Transformer V2 on the right. After each main block of ResNet50, the ResNet features are adaptively fused with the Swin Transformer V2 features. The fused representations are propagated through the subsequent ResNet blocks, while the Swin feature map is gradually refined through updates.

When training deep neural nets, there comes a point where the accuracy does not improve or deteriorate with increasing depth despite adding more layers. This phenomenon is not due to overfitting, as adding more layers paradoxically increases the training error [39]. This is known as the degradation problem. To solve this problem, He et al. [39] introduced an architecture named Residual Network (ResNet). ResNet has been proposed in different depths, such as ResNet18, ResNet34, ResNet50, ResNet101, and ResNet152, where the numbers indicate the number of layers in each model. A residual block consists of a series of layers that are connected to each other by so-called shortcut connections. These connections allow activations to be passed directly from one layer to the next, improving the flow of information and the efficiency of modeling [39]. So if the current layer is not needed, it can be bypassed thanks to this identity, which reduces overfitting issues [39]. ResNet50 consists of 50 weighted layers, forming a hierarchical deep learning architecture designed for efficient feature extraction and classification. The general structure of ResNet50 is shown in Fig 2 on the left. At the beginning of the network, an input layer processes the raw image using a 7×7 convolutional layer followed by a max pooling layer. This initial stage is responsible for capturing low-level features such as edges and textures while reducing the spatial dimensions of the input, thereby increasing computational efficiency [39]. Following the initial convolutional layer, the network is divided into four sequential blocks, each containing a varying number of bottleneck blocks. These four stages are designed to progressively refine feature extraction, transitioning from basic texture recognition to more abstract and complex representations of objects. At the end of the final block, GAP is applied, reducing the spatial dimensions to a single feature vector per channel. This is followed by a fully connected layer, which performs the final classification task. The structure of ResNet50 allows it to efficiently learn increasingly abstract representations, making it a powerful architecture for image classification and feature extraction [39].

#### Swin Transformer V2.

Swin Transformer V2 (Large version) is a scalable and high-performance vision transformer architecture optimized for processing high-resolution images [40]. Unlike traditional CNNs, Swin Transformer V2 employs a hierarchical self-attention mechanism with shifted windows (Shifted Window Self-Attention), enabling efficient processing of large images while reducing computational complexity [41]. The input image is divided into non-overlapping 4×4 patches, which are processed through a patch embedding layer with 192 channels; the hierarchical architecture progressively increases the number of channels while reducing spatial resolution, while the shifted window mechanism connects adjacent regions to enable efficient contextual modeling [40]. The self-attention operation for a given window is mathematically defined as:


$$Attention(Q,K,V)=Softmax(\frac{QK^{T}}{\sqrt{d}}+B)V$$ (10)

where *Q*,*K*,V∈${\mathbb{R}}^{M^{2}\times d}$ represent the query, key, and value matrices, respectively, derived from the input features, the term *d* denotes the dimensionality of query/key, while *B* ∈ ${\mathbb{R}}^{M^{2}\times M^{2}}$ is the relative position bias term for each head; and *M*^2^ is the number of patches in a window [41]. The shifted window design facilitates interactions between adjacent regions, enabling the model to efficiently capture contextual information across the entire image while preventing an exponential increase in computational complexity [41]. A distinction from conventional Vision Transformers is the Shifted Window Attention mechanism. Instead of applying computationally expensive global self-attention to the entire image, Swin Transformer partitions the image into small, overlapping windows, within which self-attention is computed locally [41]. To enhance training stability in very deep models, Swin V2 employs a post-normalization technique, where normalization is applied after the residual connection [40]. Additionally, Swin V2 introduces a logarithmic window size adjustment, enabling the model to automatically select larger windows for high-resolution images, thereby improving computational efficiency [40]. Compared to its predecessor, Swin V2 also incorporates a quantized Relative Position Bias (RPB), which reduces memory consumption while preserving precise positional information [40]. The Swin Transformer’s ability to model long-range dependencies is particularly advantageous for zipper defect detection because the occurrence and severity of defects are often influenced by their spatial context. By capturing global relationships and integrating information across the entire image, the model gains a more comprehensive understanding of structural patterns. This allows for the more accurate detection and differentiation of defects that vary in size, shape, and appearance, which ultimately improves the robustness.

#### Squeeze-and-Excitation (SE) block.

**Fig 3:**
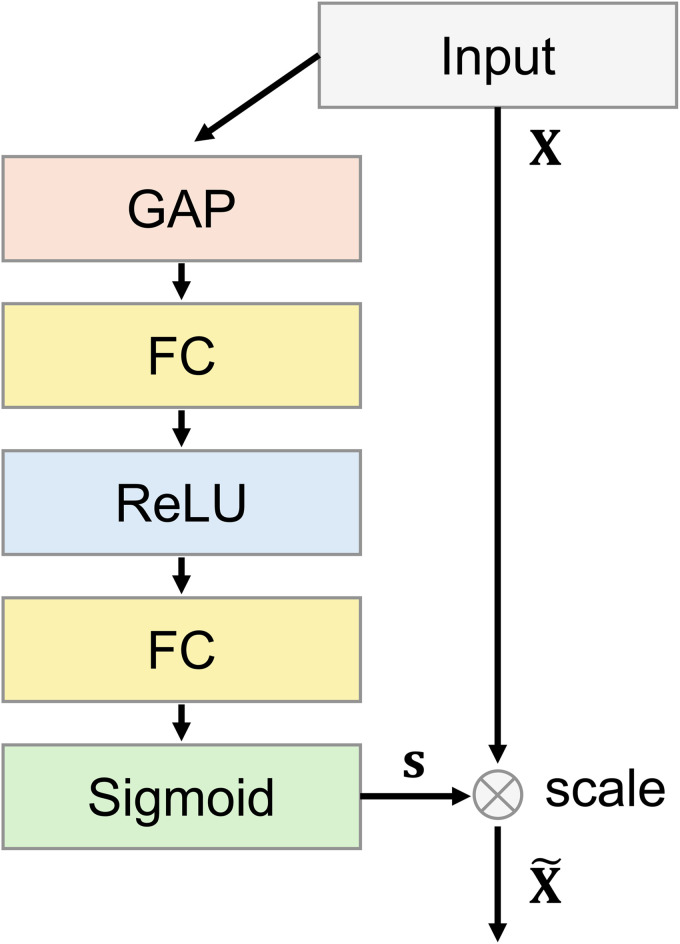
Illustration of the Squeeze-and-Excitation block [38] integrated into the AFPN.

The SE block introduced by Hu et al. [38], which can be seen in Fig 3, aims to improve the quality of the representations generated by the neural network. The SE block is designed to help the network focus on relevant features and suppress less relevant ones. The block consists of two main operations, the squeeze and the excitation. In the squeeze operation, the information of the feature map (height *H*, width *W*) per channel *x*_*c*_ is compressed to one scalar value *z*_*c*_ by global average pooling [38].


$$z_{c}=\frac{1}{H\times W}\sum_{i=1}^{H}\sum_{j=1}^{W}x_{c}(i,j)$$ (11)

This is followed by the excitation operation, in which the result from the squeeze operation is processed by a small neural network that calculates a weight between 0 (unimportant) and 1 (very important) for each channel. This small neural network consists of two FC layers. The first fully connected layer is followed by a ReLU activation and the second by a sigmoid function, which scales the values between 0 and 1 to produce a 1D vector **s** that is multiplied with the original feature map **X** to generate a scaled feature map $\tilde{\text{X}}$ [38].


$$\text{s}=\sigma ({\text{W}}_{1}\;ReLU({\text{W}}_{2}\;\text{z}))$$ (12)

$$\tilde{\text{X}}=\text{s}\text{X}$$ (13)

Here, **W**_**1**_, **W**_**2**_ ∈ ${\mathbb{R}}^{C\times \frac{C}{r}}$ are the weight matrices of the FC layers with *r* as reduction factor.

### Process of training

**Fig 4:**
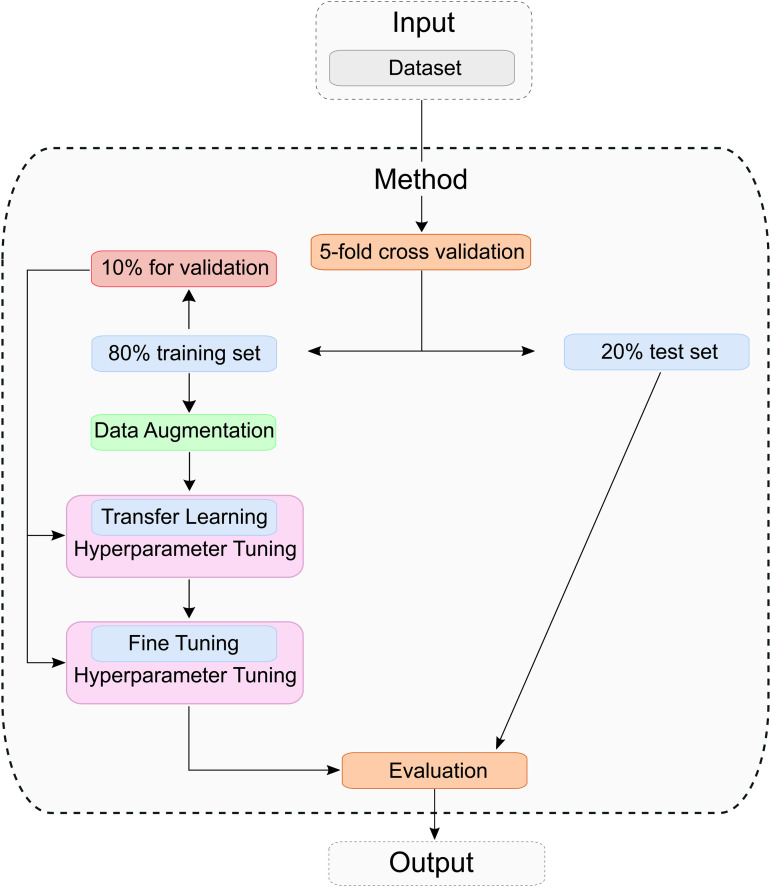
Training and Evaluation approach: The dataset is split into a training set and a test set. Data augmentation is applied to the training set, followed by model training using transfer learning and fine-tuning. Finally, the resulting model is evaluated on the test set.

A schematic representation of the training process is provided in Fig 4. Before training the model, a stratified 5-fold cross-validation from the scikit-learn library [42] was used to divide the entire dataset into five equal parts. Cross-validation is a robust and reliable method for evaluating a model. Thus, each part contains 1/5 of the total data. For the training and evaluation process, four parts (80% of the data) are used to train the model, and one part (20% of the data) is used for the subsequent evaluation. A stratified 5-fold cross-validation was explicitly chosen to ensure that each fold of the dataset has the same proportion of classes. This is not the case with normal k-fold cross-validation (with k∈ℕ and *k* > 1) and can lead to biased results with rebalanced data if one or more folds contain data of a particular class. This procedure is repeated five times. To prevent data leakage and ensure strict image independence between the training and test sets, cross-validation was performed at the image level using non-overlapping indices. This ensures that each image appears in exactly one subset (training, validation, or testing) per a fold. No augmentation or preprocessing was performed before splitting, so no even slightly altered images do appear across the subsets. This prevents overly optimistic tests and enables a methodologically rigor assessment of model performance. We have chosen this split ratio (*k* = 5) because it represents a balanced relationship between performance and computational effort. In our training and evaluation process, an additional 10% of the training data per fold is selected for validation and hyperparameter tuning.

In each fold, the images are pre-processed for training, validation, and testing, so all images are scaled to a resolution of 192×192 px with three RGB color channels. To address the imbalance between classes, the class weights are integrated into the loss function. Furthermore, all images are subjected to normalization, with the mean set to [0.485, 0.456, 0.406] and the standard deviation to [0.229, 0.224, 0.225]. Additionally, the decision was made to augment the training dataset by employing data augmentation techniques, including RandomRotation(), RandomAffine() and RandomResizedCrop(). These techniques were chosen to ensure adaptability to various image representations and to effectively mitigate the risk of overfitting. Images may be scaled differently, or the image content may vary in position. These techniques simulate these scenarios and are intended to reduce the model’s sensitivity to such variations. In this study, the limits of the parameters were established with the objective of ensuring that the resulting maximum value would not be excessively high. The maximum value for random rotation is set at ± 10°, and the cropping interval is constrained to [0.9, 1.0], indicating that no more than 10% of the image can be trimmed. Random shifts in the x and y directions of the image are possible up to ± 10%. The total number of epochs is set to 100 for the training of the model. The batch size during the transfer learning phase is set to 16.

The dual architecture undergoes two fundamental training phases in each fold: transfer learning and subsequent fine-tuning. In both phases, hyperparameter tuning is performed to determine the optimal set of hyperparameters for the model in each fold, and AdamW [43] is used as the solver for each training. The specific options considered for the Bayesian optimization are presented in Table 2, with distinct hyperparameters employed for each phase. For hyperparameter optimization, the Optuna package is utilized. The optimization process is based on the Tree-Structured Parzen Estimator (TPE), a Bayesian optimization method. Throughout the optimization process, 20 trials are conducted to identify the hyperparameters that result in the lowest validation loss. Each trial is allowed to run for a maximum of 100 epochs. Furthermore, the early stopping callback is implemented, ensuring that if the validation loss does not improve for ten consecutive epochs, the training process is automatically terminated to prevent overfitting and unnecessary computational overhead. During the transfer learning phase, all feature extraction layers are frozen, while the fully connected layers remain trainable. This approach allows the model to gradually adapt to the classification task while leveraging the pre-trained ImageNet weights [44].

**Table 2:** Overview of the hyperparameters used for hyperparameter tuning. TL = Transfer learning; FT = Fine-Tuning.

Hyperparameter	Minimum Value	Maximum Value	Step	TL or FT
Dropout	0.1	0.5	0.05	TL, FT
Learning Rate (1)	10^−4^	10^−2^	calculated logarithmically	TL
Learning Rate (2)	10^−6^	10^−4^	calculated logarithmically	FT
Weight Decay	10^−5^	10^−3^	calculated logarithmically	TL, FT
Batch Size	–	–	8, 16, 32	FT

The hyperparameters, along with their ranges, correspond to their functions in the training process. The dropout rate lies between 0.1 and 0.5 The lower limit was selected as a minimum to prevent overfitting, while the upper limit of 0.5 represents a compromise between substantial reduction in overfitting and considerable information loss. In the context of transfer learning, where only the custom classifier head is trained from scratch, larger learning rates can be employed, as there is no requirement to preserve pre-trained weights. Additionally, the randomly initialized weights must be swiftly adjusted towards the optimal weights. Therefore, during this stage, a rate between 10*e*^−4^ and 10*e*^−2^ is selected. Lower rates (10*e*^−6^ and 10*e*^−4^) are employed for fine-tuning, thus mitigating too heavy an impairment to the pre-trained feature representations, and instead facilitating their adaptation to the domain. Weight decay is instrumental Weight decay controls L2 regularization and thus plays a key role, especially in large models. As a balance between over-regularization and under-regularization, which can lead to unstable tuning, a decay between 10*e*^−5^ and 10*e*^−3^ is chosen. Once the optimal hyperparameters have been identified, a final transfer learning model is trained using these parameters, following the same training configuration as during the optimization process. The model achieving the lowest validation loss is stored and subsequently utilized as the base model for the subsequent fine-tuning phase. During fine-tuning, the hyperparameters specified in Table 2 are optimized. In contrast to the transfer learning phase, additional feature extraction layers are unfrozen in addition to the fully connected layers, allowing the model to further adapt to the new domain. Analogous to the transfer learning phase, once the optimal hyperparameters have been determined, a final fine-tuned model is trained. The model with the lowest validation loss is stored as the final version. After completing this step, the model undergoes an evaluation. The performance metrics are computed using the corresponding test subset, which comprises 20% of the dataset. This evaluation employs common functions such as *accuracy*_*score*() and *f*1_*score*() from the scikit-learn library. These functions are used to assess various aspects of the model’s performance. All relevant metrics are computed and stored for each fold in the cross-validation process. At the conclusion of this process, the average values of all five folds are calculated and reported, providing a comprehensive summary of the model’s overall performance.

### Evaluation metrics

To evaluate and interpret the model’s performance, we employ the following performance indicators: Accuracy, Balanced accuracy, True Positive Rate (sensitivity, recall or TPR), True Negative Rate (Specificity or TNR), Positive Predictive Value (precision or PPV), Negative Predictive Value (NPV), Cohen’s Kappa, F1-Score and at least Area Under the (ROC-)Curve (AUC). The accuracy determines the overall effectiveness of a model [45]. To mitigate inflated performance estimates on imbalanced datasets, balanced accuracy can be utilized, defined as the arithmetic mean of sensitivity and specificity [46,47]. The TPR metric indicates the accuracy of classifying the positive class, and maximizing it increases the likelihood of correctly identifying true members of the positive class [48]. The TNR, on the other hand, indicates how effectively a classifier identifies negative classes [45]. The PPV metric assesses prediction accuracy for the positive class by indicating the proportion of positive predictions that correctly match true positive instances [48]. The NPV, equivalent to the precision for the negative class, is the ratio of correctly classified negative samples to all samples classified as negative [49]. Cohen’s kappa describes the reliability of a model by measuring the agreement between two judgments. It ranges from −1–1, where a Cohen’s kappa of −1 indicates complete disagreement, and a Cohen’s kappa of 1 signifies perfect agreement [50]. The harmonic mean of precision and recall, called F1-Score, ranges from 0 to 1, with the minimum (0) occurring when all positive samples are misclassified (true positives = 0) and the maximum (1) occurring for perfect classification (false negatives = false positives = 0) [51]. AUC means Area Under the (ROC)-Curve. The ROC curve, obtained by plotting sensitivity against the false positive rate at all possible threshold values, is a monotonically increasing function within the unit square from (0, 0) to (1, 1), where closer proximity to (0, 1) indicates better predictions [52]. AUC, a scalar number between 0 and 1 representing the expected performance of the ROC curve (with 1 being the best) has the important statistical property of equating to the probability that the classifier will rank a randomly chosen positive instance higher than a randomly chosen negative instance [53]. In this study, the following notation was used: defect-free images belong to the negative class, while images with defects belong to the positive class.


$$Accuracy=\frac{TP+TN}{TP+TN+FP+FN}$$ (14)

$$Balanced\;Accuracy=\frac{TPR+TNR}{2}$$ (15)

$$PPV=\frac{TP}{TP+FP}$$ (16)

$$NPV=\frac{TN}{TN+FN}$$ (17)

$$TPR=\frac{TP}{TP+FN}$$ (18)

$$TNR=\frac{TN}{TN+FP}$$ (19)

$$Cohen^{\prime }s\;Kappa=\frac{P_{0}-P_{e}}{1-P_{e}}$$ (20)

TP = True Positives
TN = True Negatives
FP = False Positives
FN = False Negatives
TPR = True Positive Rate
TNR = True Negative Rate
P_0_ = Observed Agreement
P_*e*_ = Expected Agreement

### Dataset

**Fig 5:**
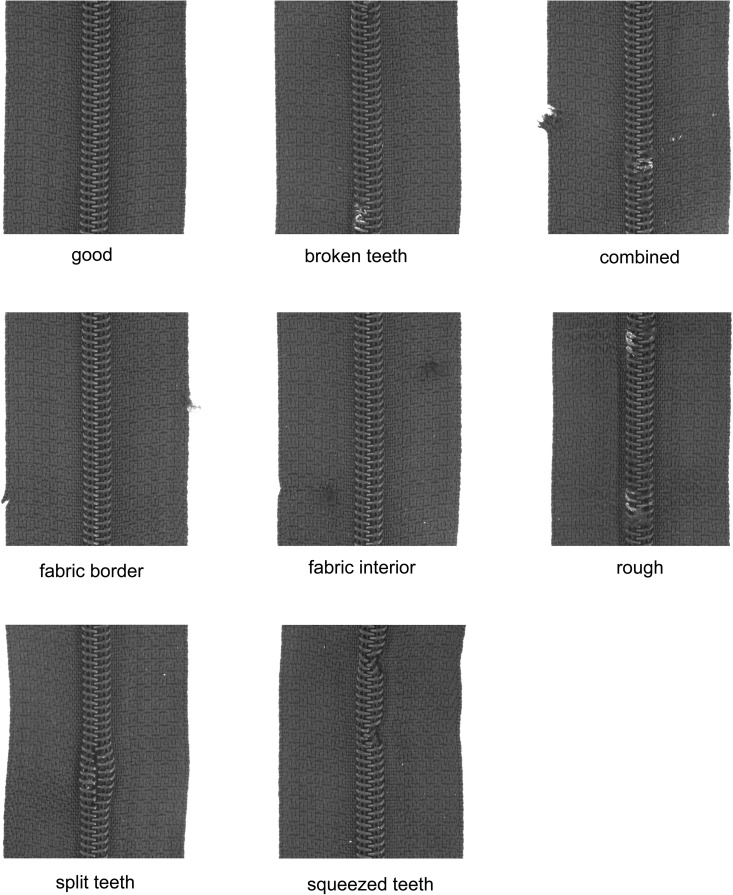
Sample images from each class found in the zipper test set from MVTec [13].

In this study, the MVTec anomaly detection dataset [13] is used. It is a benchmark dataset focused on real-world industrial inspection that contains high-resolution images of different manufactured products. The dataset consists of 15 classes of objects and textures and includes a total number of 5,354 unannotated color images of 15 different objects in the range between 700×700 px and 1024×1024 px. Of these, 3,629 images are intended for training and 1,725 images for testing. The training set contains only images without any anomalies, but the test set contains both normal and abnormal images and additional anomaly ground truth mask labels for segmentation evaluation. For the zipper task, the dataset consists of a total of 391 images, 240 for the training and 151 for the test. Of the 151 images in the test set, 32 are defect-free, as they are assigned to the class *good*, and 119 are defective. The test folder is divided into: Broken teeth (19), Combined (16), Fabric border (17), Fabric interior (16), Good (32), Rough (17), Split teeth (18), and Squeezed teeth (16). The numbers in brackets indicate how many images of the respective test classes are available. All images in this dataset have a resolution of 1024×1024 pixels. The categories of the dataset are shown in Fig 5.

### Setup

For training and testing the architecture, an NVIDIA L40S GPU with 48 GB memory with PyTorch 2.5.0 is used. Furthermore, Python version 3.11.7 and CUDA version 12.4.1 were used. The architecture was trained using the AdamW [43] solver for a maximum of 100 epochs in various phases, such as transfer learning and fine-tuning. To identify the optimal parameters for transfer learning and fine-tuning, hyperparameter tuning was performed in 20 trials using Optuna (version 4.2.1). To avoid overfitting and save computation time, the callback function *early stopping* with a *delta* = 0.001 was used, which stops the training after 10 consecutive epochs in which the validation loss has not decreased. Scikit-learn (version 1.5.2) was utilized for stratified cross-validation and the computation of performance indicators. Throughout the entire training and validation process, the images were converted to a resolution of 192×192 pixels.

## Results

Our approach has led to the results displayed in Table 3. DualFusionNet was evaluated through the stratified 5-fold cross-validation technique. The architecture was evaluated in terms of accuracy, TPR, PPV and Cohen’s Kappa. Additionally, the F1-score, balanced accuracy, TNR, NPV and AUC are reported. Table 3 shows the results of DualFusionNet. This table contains five different models of DualFusionNet, each of which was trained and tested with different data.

**Table 3:** Performance of DualFusionNet over five cross-validation runs. All metrics are reported as percentages, except for the Kappa value.

Metric\Fold	1	2	3	4	5	AVG
Accuracy	100	100	100	98.72	100	**99.74**
Balanced Accuracy	100	100	100	97.92	100	**99.58**
TPR	100	100	100	95.83	100	**99.17**
TNR	100	100	100	100	100	**100**
PPV	100	100	100	100	100	**100**
NPV	100	100	100	98.18	100	**99.64**
Kappa	1.0	1.0	1.0	0.9696	1.0	**0.9939**
F1	100	100	100	97.87	100	**99.57**
AUC	100	100	100	100	100	**100**

Additionally, Table 4 presents a comparison of the proposed architecture with other architectures, including ResNet50, the Swin Transformer and a DualFusionNet variant with AFPN, but without the use of an SE block. Compared to the standard architectures, DualFusionNet demonstrates improvements across most performance indicators. However, exceptions include TNR and PPV, where no improvements are observed. DualFusionNet achieves 99.74% in accuracy, an increase of 0.51 percentage points (pp) over the second-best result. Furthermore, the increased TPR of 99.17% is to be highlighted, which plays a special role in the quality sector, as it indicates in this case whether a defective zipper is really defective.

**Table 4:** Comparison of DualFusionNet with the separate architectures ResNet50 and Swin Transformer V2, as well as DualFusionNet using AFPN but without using the SE block. All results are the average of five runs from the cross-validation.

Metric	DualFusionNet	DualFusionNet (AFPN only)	ResNet50	Swin Transformer V2
Accuracy	**99.74%**	99.23%	97.95%	98.21%
Balanced Accuracy	**99.58%**	98.75%	96.60%	97.26%
TPR	**99.17%**	97.50%	93.19%	94.89%
TNR	100%	100%	100%	99.64%
PPV	100%	100%	100%	99.20%
NPV	**99.64%**	98.92%	97.19%	97.89%
Kappa	**0.9939**	0.9816	0.9497	0.9564
F1-Score	**99.57%**	98.70%	96.39%	96.90%
AUC	**100%**	99.98%	99.63%	99.78%

**Fig 6:**
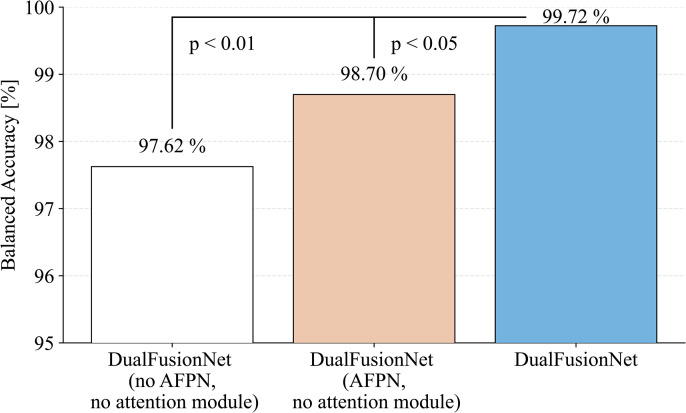
Comparison of balanced accuracy across three DualFusionNet variants: (i) without AFPN and attention mechanisms, (ii) with AFPN but without attention, and (iii) the full DualFusionNet architecture. Results are averaged over 3 runs with 5 folds each (*n* = 15). Both reduced variants show statistically significant lower performance compared to the full DualFusionNet, as shown by a paired Wilcoxon signed-rank test: without AFPN and attention (*p* < 0.01) and with AFPN but without attention (*p* < 0.05). The mean balanced accuracy increases from 97.62% and 98.70% to 99.72% in the full architecture.

In addition, we evaluated the impact of the architectural extensions in DualFusionNet using statistical significance tests. The results are shown in Fig 6. DualFusionNet was compared with DualFusionNet without AFPN and without attention module and with DualFusionNet with AFPN without attention module over a total of 15 folds (3 runs with 5 folds each). The architecture we proposed achieves a balanced accuracy of 99.72% on average, while the baseline variant achieves only 97.62%, the AFPN variant achieves 98.70%. To perform a paired statistical comparison, a Wilcoxon signed-rank test was conducted, which confirmed that the improvement is statistically significant (p-value = 0.0056 and p-value = 0.0222) in both cases. These results show that it is precisely the architectural innovations we proposed that bring about the statistically significant performance advantage.

**Fig 7:**
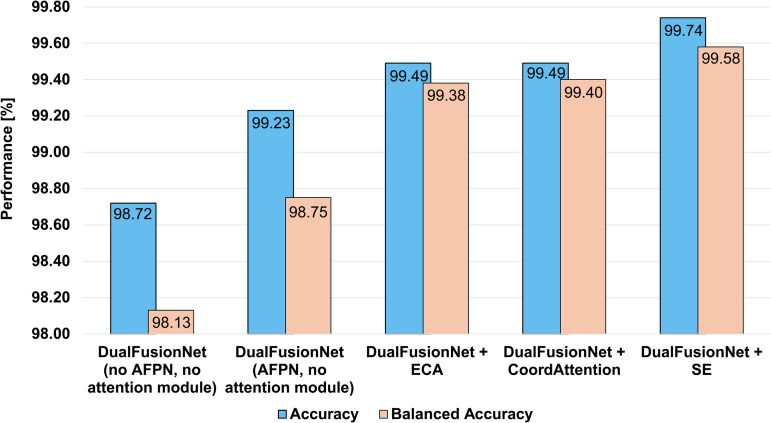
Comparison of effectiveness through the use of attention modules (Efficient Channel Attention (ECA), CoordAttention and Squeeze-and-Excitation (SE)) integrated into AFPN. It is evident that the use of an attention module in AFPN improves performance. Furthermore, the SE block achieves the highest performance in terms of accuracy and balanced accuracy.

**Fig 8:**
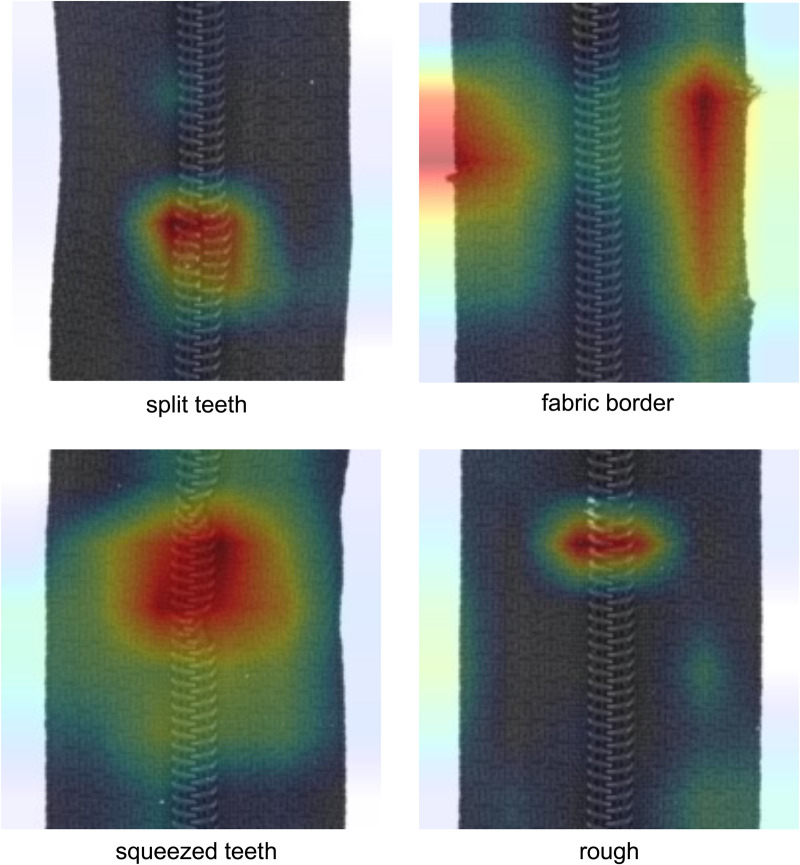
Grad-CAM heatmaps of four different zipper defects [13], showing that DualFusionNet focuses on the correct regions with defects in the image.

To demonstrate the effectiveness of AFPN and the integrated attention module in AFPN, Fig 7 shows a comparison of the effectiveness of the various attention modules. As can be seen, the variant without AFPN or an attention module achieves the lowest performance. Additionally, the use of an attention module in AFPN significantly improves performance, with SE achieving the highest values. Fig 8 shows four Grad-CAM heatmaps of four different defects, with DualFusionNet focusing on the important regions.

**Fig 9:**
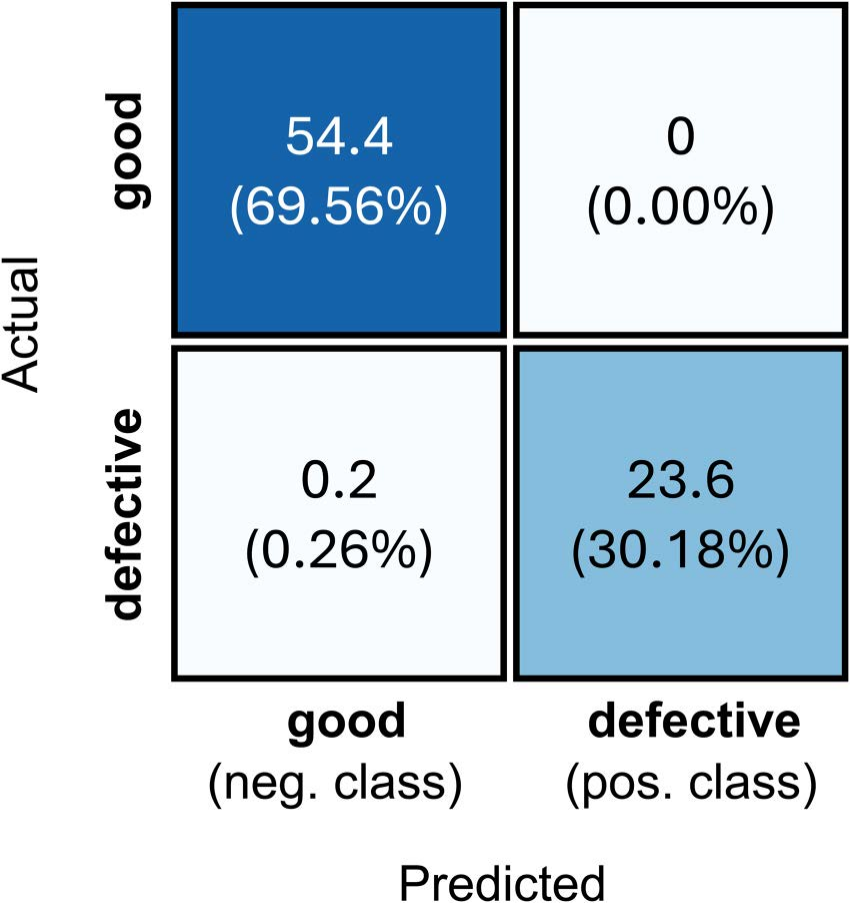
Averaged confusion matrix of the proposed architecture DualFusionNet.

The averaged confusion matrix in Fig 9 shows that DualFusionNet is excellent in detecting non-defective zippers and also delivers very good results in detecting defective zippers.

## Discussion

### Performance analysis

**Fig 10:**
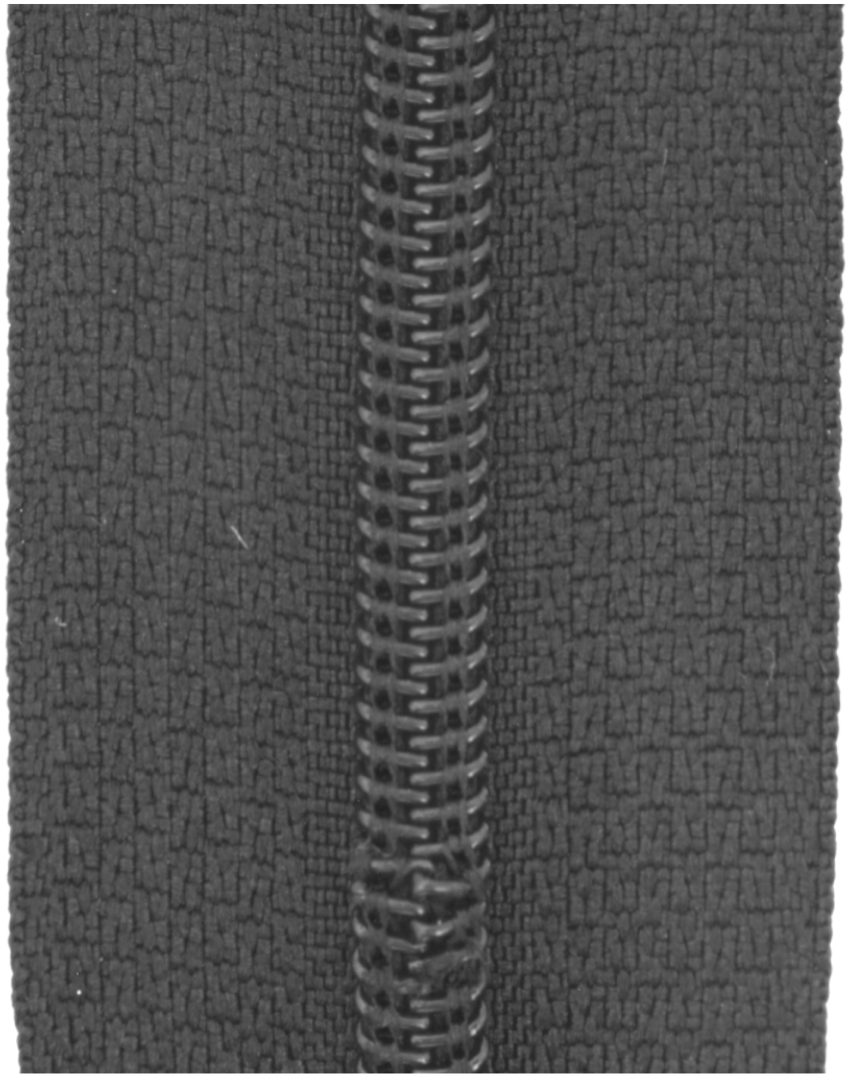
Example of a misclassified zipper image [13] with subtle split-teeth defects in the lower region.

For a comprehensive assessment of the architecture’s performance, a detailed examination of model failures and robustness can be carried out first, followed by an examination of the individual aspects of the architecture. The error analysis shows that the model’s misclassifications were limited to borderline cases with more subtle defects. Fig 10 shows an example of a “bad” sample that was incorrectly classified as “good.”

Especially in conjunction with the high TNR and the structure shown in GradCAM Analysis, this demonstrates that the model does not make systematic misclassifications, but rather fails in borderline cases that are visually difficult to distinguish. This also has implications for the model’s practical robustness, as the resulting errors are near-threshold cases and do not represent a lack of stability or general reliability. Even though the model integrates global and local features in order to perform classification as robustly and efficiently as possible, the model’s practical robustness can be more challenging in practice due to noise and lighting variations than is reflected in this error analysis.

The results, as shown in Table 4, demonstrate that DualFusionNet achieves the highest values in most performance metrics compared to the other architectures listed in the table. The ResNet50 architecture was used to extract specific local features such as edges, textures, and small, fine defects. However, as shown in Table 4, ResNet50 consistently achieves the lowest performance metrics, except for TNR and PPV, where it outperforms the standard Swin Transformer V2. As the second component of DualFusionNet, the Swin Transformer V2 was selected. Swin Transformer V2 is able to capture both local and especially global context information within an image. This is particularly advantageous for the detection of defects with varying shapes and sizes, which cannot be effectively detected with only 3×3 convolutions. The results indicate that Swin Transformer V2 outperforms ResNet50 across almost evaluation metrics, except for TNR and PPV, suggesting that global contextual relationships play a more significant role than purely local feature extraction in defect detection within zipper images. The combination of both architectures shows an improvement in accuracy and balanced accuracy. This shows that the combination of local and global features is beneficial. These characteristics can not only bring measurable performance advantages, but also be conceptually beneficial when used in practice. If possible, our deep learning model could be combined with traditional inspection methods in order to improve overall flaw detection accuracy. Traditional, mostly rule-based methods, e.g., for edge detection or measuring image statistics, have strengths when dealing with clearly defined, well-localized error types. Deep learning is mostly used to detect complex defects. The ability to detect both local and global defects and, if necessary, errors that are only visible in the interaction between these two perspectives is of particular importance, as this closes a gap that is not covered by traditional inspection methods or simpler deep learning networks.

In this dual variant, only the output of both architectures was fused, which then passed through two FC layers, with a dropout layer between the FC layers. However, adding AFPN further increases performance, demonstrating that merging the outputs of separate architectures is suboptimal. DualFusionNet (AFPN only) improves performance compared to the variant without AFPN by 0.51 pp in accuracy and 0.62 pp in balanced accuracy (corresponding to a relative error reduction of 39.84% and 33.15% respectively). These results suggest that systematically fusing individual features from ResNet50 and Swin Transformer V2 and reintegrating them into their respective architectures is more effective. This approach enables a more efficient processing of both local and global relationships, thereby maximizing the advantages of each architecture. As Fig 7 shows, there are variants of DualFusionNet with AFPN and various attention modules, as well as AFPN without attention modules and a plain variant. Integrating attention modules into AFPN appears to be a very advantageous way to further increase performance. Variants of DualFusionNet with ECA and CoodAttention achieve a 0.2 pp increase in accuracy, corresponding to a 33.77% relative error reduction. Using SE achieves the best results, with 99.74% and 99.58% accuracy and balanced accuracy, respectively. Compared to using ResNet50 and Swin Transformer V2 separately, this corresponds to an accuracy increase of 1.79 pp and 1.53 pp, respectively. Using DualFusionNet results in fewer errors by 87.32% and 85.47%, respectively. In addition to balanced accuracy, TNR, and PPV. TPR and NPV play a crucial role in defect detection. This metric is particularly important in quality control to ensure that defective products are not mistakenly classified as defect-free. NPV measures the reliability of negative predictions by assessing how often instances predicted as negative are truly negative. The proposed architecture achieves the highest NPV value of 99.64%, making it highly suitable for quality control in zipper manufacturing.

In terms of the impact of DualFusionNet on the current quality control of zippers, the architecture, if implemented in manufacturing, can enhance the process by ensuring automated and objective error detection. The high TPR and NPV are particularly relevant in production environments, as they reduce the likelihood of errors being overlooked and thus lower rework rates. The system can be integrated into existing camera systems, either as a replacement for previous visual inspections or as a preliminary inspection.

### Contextualization within existing literature

Upon examination of Table 1 from section Related Work, it becomes evident that commendable outcomes were attained in the studies that also addressed the issue of defect detection in zippers. Nevertheless, there are notable differences between our work and the work carried out previously. Similarly, Fraccaroli et al. [10] employed the MVTec dataset for the purpose of detecting defects in zippers. However, their focus was limited to the detection of defects in the zipper tape, which resulted in three out of the seven available defect classes being relabeled as non-defective. Additionally, the authors conducted a train-test split for evaluation purposes, with the test set comprising 45 images. In contrast, our approach involves utilizing all defect classes from the dataset, thereby introducing a more challenging scenario for the model. Furthermore, the authors assessed their methodology utilizing an industry dataset that is not publicly accessible. The authors did not offer any additional insights due to a non-disclosure agreement (NDA). Nevertheless, we were able to achieve a higher accuracy (+4.18 pp) with our approach on the same dataset.

In the study by Buettner et al. [16], the MVTec dataset is also utilized, allowing for a direct comparison with our results. The study employs five different architectures, applying both transfer learning and fine-tuning, with a primary focus on the use of a Gaussian filter. The best-performing model in terms of accuracy (95.92%) and balanced accuracy (93.97%) is VGG19 when utilizing the Gaussian filter. In comparison, the hybrid architecture presented in this study achieves superior results (Accuracy: + 3.82 pp; Balanced Accuracy: + 5.61 pp), demonstrating that this approach provides a more accurate classification. Xu et al. [37] employ a non-public dataset derived from production to identify surface defects. Due to the dataset imbalance, oversampling is used. The number of defect classes is not specified; only three example types are provided. For evaluation purposes, 80% of the data is used for training and the remainder for testing. Given the dataset, which contains a greater number of images than MVTec, in addition to differing evaluation methodologies, direct comparison with our results is challenging. Looking only at the PPV (98.98%), F1 (98.98%), and TPR (98.98%) indicators, our dual architecture achieves higher values.

In both works [[10,37]], the same procedure is employed, namely a train-test split for evaluation. When this procedure is applied only once, however, it provides a biased assessment of the model’s performance. In contrast, in our work, a stratified 5-fold cross-validation is used to evaluate the robustness of the model. The use of stratified k-fold cross-validation also ensures that the proportions of the classes are preserved, thereby avoiding the potential issue of unequal splits that can arise with the train-test split approach unless it is defined.

The remaining two research papers [5] and [4], focused specifically on the identification and classification of various types of defects in zippers. As a result, the detection of zippers that were free from defects was not within the scope of their investigations. In the study conducted by Fang et al. [5], the researchers employed an industry-specific dataset, the origins of which are unknown. To enhance the robustness and comprehensiveness of their study, they used data augmentation techniques to expand this dataset significantly. Originally comprising 918 images, the dataset was increased to 18,351 images. Using their method, they achieved a mean average precision of 99.52%.

Sun et al. [4] used the MVTec dataset in their study, employing a number of data augmentation techniques to artificially increase the number of images, with their dataset ultimately consisting of 2,706 images. Similar to [5], the authors focused exclusively on the classification of defects that occur during zipping, achieving 100% accuracy for four defect classes and 99% accuracy for two defect classes using the YOLOV5 algorithm. Furthermore, our work diverges significantly from that of [5] and [4] in several key aspects. Specifically, our research focuses on binary defect detection, which is distinctly different from the identification of individual defect types. In our study, we categorize zippers into only two groups: defective and non-defective. This binary classification is crucial for quality control, as it simplifies the analysis by not prioritizing the identification of specific defect types. Additionally, we utilize the MVTec dataset in its original form without artificially expanding it, maintaining its integrity for evaluation. There are also discrepancies in the evaluation methodologies used. For instance, in the study by Sun et al. [4], a single train-test split is employed with 150 images per class designated for testing. In contrast, we adopt a stratified 5-fold cross-validation approach. This method is more robust and comprehensive, providing a deeper understanding of the model’s reliability and performance across different data subsets.

In summary, the dual architecture DualFusionNet proposed in this study proves to be an efficient approach for classifying defective and non-defective zippers. The high TNR and NPV confirm the reliability of defect detection, which is particularly crucial in quality control applications. The values presented here represent a significant improvement compared to the manual quality control that is currently still practiced. For companies that still have a manual control process, the transition to an automated quality control system seems to be an economically viable next step, as this would allow for faster and more efficient quality control, leading to cost savings. In addition, it is also conceivable to combine our deep learning model with the traditional method of inspections, for example by using classic methods as pre-filters or as downstream plausibility checks following an assessment carried out using deep learning. Such hybrid variants can reduce false negatives and generate greater robustness in borderline cases.

### Implications for labor efficiency

The potential labor savings of automated defect detection can be estimated from numbers reported in the textile inspection literature. Manual quality inspection has been reported to achieve only 60% to 75% defect detection accuracy [54]. Inspectors can also only sustain effective visual focus for around 10–30 minutes before fatigue starts to hurt their performance [54,55]. A study on a real woven fabric production line found that manual inspection takes about 2 seconds per image at a classification rate of 75%. An automated system processed the same images in 0.125 seconds at a detection rate of 96.60%, which is a 16-fold reduction in processing time [56]. Applied to a zipper production scenario of 1,000 units per shift, manual inspection would take about 33 minutes of focused work. Given the fatigue constraints, the inspector would also need several rest breaks. This pushes the effective inspection time well beyond one hour. An automated system like DualFusionNet, which achieves 99.74% accuracy, processes the same volume in seconds without any drop in performance. Manual inspection also misses between 25% and 40% of defects [54]. The proposed approach brings that below 1%. This reduces downstream costs from rework and customer returns. For a facility running multiple shifts, this adds up to several labor hours saved per day at a much higher detection quality.

### Comparison with large vision models

To test whether large vision models (LVMs) can replace purpose-built architectures for industrial defect detection, LLaVA-1.5-7B [57], a 7-billion parameter open-source vision-language model from the LLaMA model family, was fine-tuned under the same evaluation setup. Fine-tuning was done using Quantized Low-Rank Adaptation (QLoRA) [58]. QLoRA adds small trainable matrices (rank *r* = 16, α=32) into the attention layers of the model. The base model stays frozen in 4-bit NF4 quantization. With this setup, only 42 million of the 7.1 billion parameters (0.60%) were trainable. The task was set up as a visual question answering problem. Each image was shown together with the prompt *“Is this zipper defective? Answer with Yes or No..”* The model was trained to produce the correct answer token. All experiments ran on an NVIDIA A100 GPU (40 GB VRAM).

The overall results are shown in Table 5. The per-fold results are listed in Table 6. A mean AUC of 98.30% ± 2.09% shows that the fine-tuned model consistently ranked defective images above non-defective ones across all five folds. This high AUC, however, only reflects the correct ordering of the model’s internal probability scores. It does not mean the model can reliably classify images. The model’s binary decisions come from applying a fixed 0.5 threshold to these scores. This threshold is common in binary classification. It assumes, however, that the probability scores are well-calibrated for the task. In a purpose-built binary classifier, the output is trained to produce meaningful probabilities around this boundary through a sigmoid activation. In LLaVA, the scores come from the relative logits of two tokens in a large vocabulary. They are shaped by the language model’s prior over natural language. They are not shaped by the structure of the defect classification problem. Because of this, the 0.5 threshold has no guaranteed link to the model’s actual decision boundary. All classification metrics fall well below those of DualFusionNet. Accuracy reaches 68.90% ± 22.30% compared to 99.74% ± 0.51%. Balanced accuracy reaches 65.98% ± 19.95% compared to 99.58% ± 0.83%. F1-score reaches 44.48% ± 36.80% compared to 99.57% ± 0.85%. A Cohen’s Kappa of 0.308 ± 0.383 compared to 0.9939 ± 0.01 further shows the unreliability of the predictions. The standard deviations also highlight a clear difference in stability. DualFusionNet produces consistent results across all folds, while LLaVA-1.5-7B shows extreme variation with standard deviations up to 45.97%.

**Table 5:** Comparison of DualFusionNet and fine-tuned LLaVA-1.5-7B on the MVTec zipper dataset. All results are reported as mean ± standard deviation over five cross-validation folds. All metrics are reported as percentages, except for the Kappa value.

Metric	DualFusionNet	LLaVA-1.5-7B (fine-tuned)
Accuracy	99.74 ± 0.51	68.90 ± 22.30
Balanced Acc.	99.58 ± 0.83	65.98 ± 19.95
TPR	99.17 ± 1.67	58.26 ± 45.97
TNR	100.00 ± 0.00	73.70 ± 38.82
PPV	100.00 ± 0.00	57.60 ± 39.25
NPV	99.64 ± 0.73	67.17 ± 35.95
Kappa	0.9939 ± 0.01	0.308 ± 0.383
F1-Score	99.57 ± 0.85	44.48 ± 36.80
AUC	100.00 ± 0.00	98.30 ± 2.09

**Table 6:** Per-fold test results of fine-tuned LLaVA-1.5-7B on the MVTec zipper dataset. All metrics are reported as percentages, except for the Kappa value.

Metric	Fold 1	Fold 2	Fold 3	Fold 4	Fold 5
Accuracy	29.11	97.44	70.51	69.23	78.21
Balanced Acc.	47.92	95.65	52.08	50.00	84.26
TPR	95.83	91.30	4.17	0.00	100.00
TNR	0.00	100.00	100.00	100.00	68.52
PPV	29.49	100.00	100.00	0.00	58.54
NPV	0.00	96.49	70.13	69.23	100.00
F1-Score	45.10	95.45	8.00	0.00	73.85
Kappa	−0.0255	0.9367	0.0568	0.0000	0.5725
AUC	95.83	95.65	100.00	100.00	100.00

The poor results point to a basic mismatch between large vision-language models and the needs of high-precision binary industrial inspection. LLaVA-1.5-7B is a generative multimodal model. It was built for open-ended visual question answering across many types of natural images. Using it for binary defect classification means turning the task into a language generation problem. A yes-or-no decision is derived by comparing the logits of two answer tokens. This setup does not fit the task well. The size of these token logits is driven by the pretrained language model’s prior over natural language. It is not driven by the defect classification problem itself. The model’s internal representations are good enough to achieve a near-perfect AUC of 98.30%. But the resulting probability scores *P*(defective) have no stable link to the 0.5 decision boundary. In some folds, the model assigns *P*(defective) > 0.5 to nearly all images regardless of class (fold 1, TNR = 0%). In others, it assigns *P*(defective) < 0.5 to nearly all images (folds 3 and 4, TPR = 0–4%). This extreme variation between folds is a direct result of the mismatch between a generative language model and a binary classification task. It cannot be fixed through parameter tuning or more fine-tuning data alone.

Three more factors add to this problem. First, the CLIP vision encoder [59] in LLaVA-1.5-7B was trained only on natural image-text pairs. It has no knowledge of industrial surface textures or defect patterns. DualFusionNet uses pretrained ImageNet weights that provide a strong foundation for recognizing textures and edges. It is then fully adapted to zipper defect classification through a two-phase training process. Second, 391 training images are enough for transfer learning with a small, task-specific architecture. They are not enough to stably adapt even 0.60% of a 7B model’s parameters across different data splits. This shows in the extreme variation between folds across all metrics. Third, DualFusionNet benefits from Bayesian hyperparameter tuning across 20 Optuna trials per training phase per fold. That kind of systematic tuning is not feasible for a 7B parameter model.

From a practical standpoint, LLaVA-1.5-7B has 7.1 billion parameters. The standard training setup from the original paper needs 8 A100 GPUs. Running the model at full precision takes up to 24 GB VRAM [57]. This limits both fine-tuning and deployment to high-end GPU hardware. That kind of cost is not realistic for most factory settings. Cheap and fast deployment on standard hardware matters most there. DualFusionNet is a small purpose-built architecture with far fewer parameters. It runs on standard GPU hardware at a fraction of the cost. The results show that fine-tuned LVMs are not a reliable option for industrial quality control. LVMs may still be useful when zero-shot generalization across new and unseen defect types is needed. This is especially true when there is too little labeled data to train a specialized model. But for high-precision binary defect classification in production, purpose-built architectures like DualFusionNet remain the better and cheaper choice.

## Conclusion

Deep learning has emerged as a powerful tool in the field of defect detection on the surface of zippers, rapidly gaining traction for quality control in various industrial applications. The objective of this study was to develop a robust and efficient model for automated quality control in zipper production. To this end, we designed DualFusionNet, a dual architecture, integrating ResNet50 and Swin Transformer V2, enhanced with an AFPN and SE block. To ensure robustness and reliability, a stratified 5-fold cross-validation approach was employed. Data augmentation techniques, including random rotation, random affine and random cropping, were applied to introduce variability and enhance the dataset’s generalization capability by improving the model’s ability to handle defects in varying positions. With this approach, the proposed architecture achieves an average accuracy of 99.74% and a balanced accuracy of 99.58%. Due to the high value observed in balanced accuracy, the implementation of this architecture proves to be a promising and effective approach. Furthermore, DualFusionNet performs well across other metrics, achieving a TPR of 99.17% and a TNR of 100%. The proposed hybrid architecture demonstrates its ability to accurately detect defects while maintaining low false-positive and false-negative rates. Thus, the architecture is suitable for quality control in industrial zipper manufacturing. This approach can also contribute to cost savings, as poor quality zippers are better identified and rejected, resulting in fewer customer complaints and less reputational damage.

### Limitations

This study introduced DualFusionNet, a novel and high-performing dual deep learning architecture. Although the results highlight the effectiveness and robustness of the proposed approach, the limitations of the work should be considered. The dataset employed in this study includes seven predefined defect classes, which means the strong performance of the proposed architecture is currently limited to this specific set of defects. In practice, however, additional and potentially unforeseen defects may appear, and their detection accuracy falls outside the scope of this dataset. Furthermore, this study used the zipper subset of the MVTec AD dataset, which is a recognized benchmark dataset for real-world industrial inspection, but this also limits its generalizability. The significance of this study is therefore limited to zipper defects and could be evaluated more broadly in the future. This also applies, for example, to aspects of the model’s practical robustness, such as noise and lightning variations, which can occur in practice. However, our model counteracts this limitation by combining local and global features.

### Future work

For future work, we plan to validate the results of the classification models presented here using additional datasets to enhance external validity. If appropriate, the research could be extended to other defect types of zippers as well as to other fabric materials. Such an extension would allow a systematic validation of the variability and robustness of the proposed model. This would strengthen the generalizability of the approach beyond the specific defect classes considered in this study. Additionally, further testing under varying lighting conditions and distortions should be conducted. Furthermore, investigating the impact of pre-processing filters on model performance appears to be a meaningful approach, as demonstrated in [16] with the use of a Gaussian filter. Beyond zipper manufacturing, the transfer of the proposed architecture to other industrial domains such as additive manufacturing or complex signal data represents a promising direction [17,18]. Furthermore, while the present study has demonstrated that fine-tuned LVMs underperform specialized architectures on small industrial datasets, future work could investigate whether zero-shot or few-shot LVM evaluation offers complementary value in scenarios with extremely limited labeled data or highly diverse and previously unseen defect types.
